# Cartas al editor

**DOI:** 10.7705/biomedica.8712

**Published:** 2026-10-16

Lima, 8 de junio del 2026

Señores

Revista *Biomédica*

Instituto Nacional de Salud

Bogotá, D. C., Colombia

Estimado editor:

Nos dirigimos a usted para presentar un comentario crítico sobre el artículo original publicado en la revista *Biomédica*, en el volumen 45, número 3, titulado “Prevalencia y caracterización de la coinfección por el virus de la inmunodeficiencia humana en pacientes hospitalizados con tuberculosis en un hospital de referencia de Bogotá”. En dicha investigación, Puentes Ariza *et al*. evidenciaron que la coinfección por *Mycobacterium tuberculosis* y HIV constituye un problema relevante en el ámbito hospitalario, asociado con inmunosupresión avanzada y desenlaces clínicos ^1^.

Con respecto al diseño metodológico, el sesgo de selección en el muestreo es el primer factor que distorsiona los resultados. Concentrar la investigación exclusivamente en un hospital de tercer nivel genera una falsa percepción de la gravedad del problema general, dado que a este tipo de instituciones llegan los cuadros clínicos más difíciles y avanzados de la región. De este modo, el índice de coinfección obtenido se limita a describir un escenario de alta complejidad muy específico, lo que impide trasladar o extrapolar estas conclusiones al grueso de la población con diagnóstico de tuberculosis o VIH ^2^^,^^3^.

Asimismo, se identifican puntos débiles en la confirmación diagnostica y en la medición de los tiempos de espera para la detección. Llama la atención que un porcentaje definitivo de la muestra fuera catalogado mediante sospecha clínica o hallazgos por imágenes, prescindiendo de un examen microbiológico definitivo. Esta falta de corroboración laboratorial es sumamente crítica al tratar con pacientes que viven con VIH, un grupo en el que la tuberculosis rompe los patrones habituales y cursa de forma atípica, lo que retrasa un abordaje oportuno. Dado que postergar el inicio de la terapia dirigida sabotea el control epidemiológico, resulta imperativo indagar a fondo en los motivos de estos desfases cronológicos para dar un sentido real a las tasas de coinfección y mortalidad reportadas ^4^.

Por último, la naturaleza retrospectiva y el corte transversal del estudio restringen drásticamente el análisis de la información. Al depender de la lectura de archivos médicos del pasado, la fidelidad de los datos queda subordinada a la exhaustividad con la que el personal sanitario haya completado los registros clínicos en su momento. Si bien este modelo es práctico para estimar la prevalencia y proponer correlaciones estadísticas, carece de la capacidad de demostrar que un evento cause el otro. Por ende, las variables asociadas al fallecimiento de los pacientes deben interpretarse únicamente como asociaciones o nexos matemáticos, descartando cualquier conclusión de causalidad directa ^5^.

En resumen, el trabajo aporta información valiosa al describir la coinfección entre tuberculosis y VIH en un entorno de alta complejidad, pero sus conclusiones deben interpretarse con cautela debido a las limitantes metodológicas expuestas. Resulta imperativo el desarrollo de futuros estudios de carácter prospectivo, respaldados por un mayor volumen de pruebas de laboratorio que garanticen la confirmación diagnóstica y que analicen con precisión los periodos de latencia en la atención médica. Esto proveerá evidencia científica sólida para optimizar las decisiones clínicas y reestructurará las políticas de salud pública enfocadas en mitigar el impacto de esta coinfección.

Sebastián Alexander Calvo

Astrid Shantalle Camacho

Allison Marina Pachas

Facultad de Ciencias de la Salud, Universidad Privada San Juan Bautista, Lima, Perú
