# Supplementary material for: Cartas al editor
Source: Biomedica. 2026 Oct 16;46(4):680–3. [Article in Spanish] doi: 10.7705/biomedica.8712 (PMC13743073; doi:10.7705/biomedica.8712)
Supplement: Related texts

# Reply: Cartas al editor

Bogotá, D. C., 6 de agosto de 2026

Señores

Revista *Biomédica*

Instituto Nacional de Salud

Bogotá, D. C., Colombia

Estimados señores:

En primer lugar, agradecemos al grupo del doctor Calvo su análisis y comentarios de nuestro trabajo, lo que por supuesto contribuye a la construcción y aplicación de la evidencia en nuestra población.

Estamos de acuerdo en la limitación para la aplicabilidad de los resultados de este trabajo a la población general. Como lo describimos en el objetivo general del estudio, la base poblacional sobre la cual se describe la prevalencia de coinfección por tuberculosis y HIV se hizo exclusivamente en pacientes hospitalizados en el Hospital Simón Bolívar de Bogotá (Colombia), institución de alto nivel de complejidad y referencia en la atención de pacientes con HIV del sector público de la ciudad, aclarando que este es el contexto de ejercicio profesional y académico de los autores.

Contrastamos estos datos con los ya descritos sobre la población mundial, del 8 % ^1^, los datos del registro colombiano de tuberculosis, del 12 % ^2^, y de la Cuenta de Alto Costo de Colombia, del 13 % ^3^. Como resaltamos en la discusión, no se pueden generalizar estos resultados a la población en atención primaria, pero consideramos que describir el comportamiento de esta coinfección en un contexto de hospitalización en una población local, aun con las limitaciones de recursos diagnósticos y terapéuticos que podemos enfrentar en la región de Latinoamérica, contribuye a la construcción de evidencia.

En cuanto a la confirmación diagnóstica, es de aclarar que la base de búsqueda de pacientes por incluir es el registro institucional de casos con infección sospechada o confirmada por tuberculosis, obligatoria para las instituciones del país. Además, los 102 pacientes con infección por tuberculosis pulmonar o extrapulmonar de donde se basó el denominador de la proporción de coinfección por HIV solo incluyó pacientes que hubieran iniciado tratamiento antituberculoso durante su evolución, decisión que fue evaluada y aprobada por el Servicio de Infectología de la institución y que, al igual que en la práctica clínica habitual, en muchos casos se basa en criterios clínicos y radiológicos que se fueron considerados suficientes por el grupo experto en tuberculosis.

Existe una limitación adicional en el acceso a los resultados de laboratorio, pues se presentaron cambios con el prestador y el sistema de información de laboratorio durante el periodo de tiempo evaluado, lo que limitó el acceso a dicha información.

Si bien se hubiera podido excluir los pacientes sin confirmación estrictamente microbiológica, esto también hubiera generado un sesgo, considerando que, según los datos de la Organización Mundial de la Salud (OMS), aproximadamente el 64 % de 6,9 millones de casos de tuberculosis pulmonar en el mundo en 2024 contaron con confirmación bacteriológica, y que esto sigue siendo una limitación en varias regiones del mundo, incluyendo aquellas con alta carga de la enfermedad ^4^.

Además, estamos de acuerdo con la limitación inherente al diseño retrospectivo y de corte transversal del estudio y los sesgos que esto conlleva. Aclaramos, nuevamente, que los desenlaces primarios corresponden a la caracterización demográfica y clínica de los pacientes, del tipo de infección por tuberculosis y del estado de inmunosupresión, y recalcamos la importancia de describir la prevalencia de la coinfección y, además, el alto porcentaje de casos de *novo* de HIV con base en el diagnóstico inicial de tuberculosis.

De forma exploratoria, se desarrolló un análisis para encontrar posibles asociaciones entre variables de interés y desenlaces, las cuales, más que confirmar causalidad, permiten generar hipótesis. La asociación encontrada entre el conteo bajo de linfocitos T CD4+ y la tuberculosis meníngea y la mortalidad es exploratoria, y se encuentra alineada con lo reportado en la literatura ^5^^-^^7^.

También creemos necesario desarrollar investigaciones de carácter prospectivo para resolver estas y otros interrogantes clínicos, idealmente con mayor acceso a pruebas diagnósticas según lo recomendado por la OMS, en la medida en que esto permita tratar de mejor forma nuestros pacientes individualmente e impactar la salud pública de nuestra población.

Gean Carlo Puentes-Ariza^1^

Sugeich Meléndez-Rhenals^1,2^

Laura Cristina Nocua-Báez^1^

Hugo Páez-Ardila^3^

^1^ Departamento de Medicina Interna, Facultad de Medicina, Universidad Nacional de Colombia, Bogotá, D.C., Colombia

^2^ Servicio de Medicina Interna, Hospital Universitario Nacional de Colombia, Corporación Salud, Bogotá, D.C., Colombia

^3^ Servicio de Infectología, Hospital Simón Bolívar, Bogotá, D.C., Colombia

## References

[1] Joint United Nations Programme on HIV/AIDS (UNAIDS) (2022). In danger: UNAIDS Global AIDS Update 2022.

[2] Fadul-Pérez S. (2022). Protocolo de vigilancia de tuberculosis.

[3] Cuenta de Alto Costo (2025). Situación del HIV en Colombia 2024.

[4] World Health Organization (2025). Global Tuberculosis Report.

[5] Seddon JA, Tugume L, Solomons R, Prasad K, Bahr NC (2019). Tuberculous Meningitis International Research Consortium. The current global situation for tuberculous meningitis: Epidemiology, diagnostics, treatment and outcomes. Wellcome Open Res.

[6] Cecchini D, Ambrosioni J, Brezzo C, Corti M, Rybko A, Pérez M (2009). Tuberculous meningitis in HIV-infected and non-infected patients: Comparison of cerebrospinal fluid findings. Int J Tuberc Lung Dis.

[7] Stadelman AM, Ellis J, Samuels THA, Mutengesa E, Dobbin J, Ssebambulidde K (2020). Treatment outcomes in adult tuberculous meningitis: A systematic review and meta-analysis. Open Forum Infect Dis.
